# Latent Classification Analysis of Leisure Activities and Their Impact on ADL, IADL and Cognitive Ability of Older Adults Based on CLHLS (2008–2018)

**DOI:** 10.3390/ijerph20021546

**Published:** 2023-01-14

**Authors:** Change Zhu, Christine Walsh, Lulin Zhou, Xinjie Zhang

**Affiliations:** 1 Department of Management, Jiangsu University, 301 Xuefu Road, Jingkou District, Zhenjiang 212001, China; 2 Faculty of Social Work, University of Calgary, 2500 University Drive NW Calgary, Calgary, AB T2N 1N4, Canada

**Keywords:** ADL ability, IADL ability, cognitive ability, development trajectory, leisure activities, panel binary regression, latent profile analysis, older adults

## Abstract

This study aimed to research the trajectory of leisure activity and the health status of older adults and analyze the effects of leisure activity on the health status of older adults. Based on the longitudinal data of CLHLS (2008–2018), the latent growth curve model (LGCM) was used; we found that the leisure activities (LA), activities of daily living (ADL) ability, instrumental activities of daily living (IADL) ability, and cognitive ability (COG) of older adults show a nonlinear downward trend over time. Furthermore, the panel binary regression analysis is used to find that leisure activities have significant inhibitory effects on ADL disorder, IADL disorder, and cognitive impairment in the older population. In addition, by using latent profile analysis (LPA), the older population is classified into three groups according to the homogeneity of the older adults’ choice of leisure activities, namely the types of relaxation, entertainment, and intellectual-learning, respectively. Based on the classification results, the analysis of one-way ANOVA shows that the rates of ADL disorder, IADL disorder, and cognitive impairment of older adults with different types are significantly different. Moreover, the inhibitory effect of leisure activities on the rate of ADL disorder, IADL disorder, and cognitive impairment of older adults is more significant in the middle-aged and high-aged groups. Therefore, older adults should be encouraged to increase leisure activities, especially those who are middle-aged and high-aged.

## 1. Research Background

As population ageing deepens globally, understanding how to maintain the health level of the older population has become an increasing concern. China has the world’s largest elderly population, estimated to reach 488 million by 2050, accounting for 35.5% of the total population (United Nations (2012). World Population Prospects: The 2012 Revision. New York, NY, USA: United Nations Department of Economic and Social Affairs. Available online at: http://esa.un.org/unpd/wpp/index.htm accessed on 10 November 2022). At present, the average life expectancy of older people in China has increased, but the older population is generally characterized by a “living with disease” phenomena. In order to effectively address the problem of ageing, China has begun to implement the healthy China strategy (www.cpcnews.cn access on 10 November 2022), with a view to improve the overall health status of the older population, reducing morbidity and improving quality of life.

With the development of research, health is understood as not only physical health, but also mental health and cognitive ability. According to the World Health Organization (WHO), health is not only the absence of physical handicaps or diseases, but also is comprised of the normal state of physical, mental, and social adaptation [1]. Therefore, studies on health in older people must be multidimensional [2]. However, existing studies on the health status of the older population largely focus on a single dimension, either physical health or mental health; for older adults, the degree to which they are able to engage in the activities of normal daily life ability and their cognitive ability are also important indicators of health status. The activities of daily living (ADL), instrumental activities of daily living (IADL), and cognitive ability (COG) represent these functions [3]. Accordingly, this study takes ADL, IADL, and COG as indicators of the health status of older adults.

In recent years, an increasing number of researchers have begun to analyze the influence of leisure activities on the health status of older people. Leisure activities refer to the meaningful activities that individuals voluntarily engage in during their leisure time that are unrelated to making a living and remuneration [4,5]. As an essential part of older adults’ life, leisure activity is significant and can be a relatively inexpensive and accessible way to promote health. Therefore, increased scholarly attention has been paid to the relationship between leisure activities and health status among the older population. For instance, studies have found that leisure activities can promote health level [6], life satisfaction [7], subjective well-being [8], and relieve stress [9] and loneliness [10], thus affecting many aspects of life happiness [11], especially health status. Specifically, numerous studies suggests that leisure activities not only subjectively promote self-rated health status [12,13,14] but also reduce cognitive disorders [15,16,17]. Additionally, research indicates that leisure activities are effective in reducing cardiovascular disease [18,19]. Furthermore, leisure activities can be effective in preventing diseases caused by normal aging [20] or physical pain caused by a sedentary lifestyle [21]. For older people who suffer from a chronic disease, leisure activities have been found to slower the progression of disease [22]. In summary, a substantial body of research suggests that leisure activities play an essential role in the health status of older adults.

Leisure activities are classified into the following five categories of activity: mental, social, physical, productive, and recreational. The analysis and by investigated the analyzing the differences in between the effects of different types of leisure activities. Paillard–Borg (2009) found that mental activities, although not the most common choice, are the most likely to increase well-being among older adults [23]. Among five types of leisure activities, it was found that social leisure activities such as visiting friends are most likely to improve the quality of life of older people. Moreover, it has been found that informal social activities, such as participation in a club, are most likely to increase happiness [24]. Furthermore, it is suggested that cultural involvement-type leisure activities promote cognitive development through cognitive retention mechanisms [25].

To summarize, existing studies on the health status of the older population have mainly focused on the presence or absence of disease, but activities of daily living (ADL), instrumental activities of daily living (IADL) and cognition (COG) are also important indicators of physical health. Additionally, even if the older population does not have a significant physical disease, they may experience problems with inadequate ADL and instrumental ADL and cognitive decline as they grow older [26,27,28]. Therefore, it is necessary to analyze the changes in the trajectory of ADL ability, IADL ability and cognitive ability of older people. Based on the Chinese Longitudinal Healthy Longevity Survey (CLHLS) (https://opendata.pku.edu.cn/dataverse/CHADS accessed on 12 November 2022) from 2008 to 2018, this study used the latent growth curve model (LGCM) to analyze the changes in the trend of health status (ADL, IADL and COG) of the older population.

In addition, in order to improve the health status of older adults, it is necessary to analyze the impact of leisure activities on the ability of ADL, IADL, and COG of older adults. Cross-sectional data were used to study the effects of leisure activities on the physical health, mental health [29], and cognitive ability of older people [30]. However, cross-sectional data are not convincing enough to verify causality between leisure activities and health status. Furthermore, as few studies focus on the effect of leisure activities on ADL and IADL, longitudinal data can be used to analyze the impact of leisure activities on the ability of ADL, IADL, and COG of the older population.

Leisure activities are comprised of a number of different undertakings each with distinct natures. Although some research has investigated the effects of different types of leisure activities on the health status of the older population, existing studies use the extrinsic characteristics of leisure activities to classify leisure activities, and the number and type of specific classifications of leisure activities are often determined by the subjective judgment of the researcher, which lacks sound theoretical evidence. To overcome this deficit, a latent profile analysis (LPA) is used to classify the older population according to their response patterns to leisure activities and to determine whether there is a significant difference in the effect of different types of leisure activities on the health status of older adults.

Therefore, this research study aims to determine the changing trajectory of leisure activities and health status, as measured by ADL, IADL, and COG, of the older population over time and determine the influence of leisure activities on the health status of this population. Additionally, a latent profile analysis of older adults based on their choice of leisure activities will be conducted.

## 2. Methods

### 2.1. Data Sources

Data for this article were obtained from the China follow-up survey on the health impact factors of older adults (Chinese Longitudinal Healthy Longevity Survey, CLHLS) of 2008, 2011, 2014, and 2018, conducted with people over 60 years of age [31]. The respondents were all interviewed face to face. The main focus of the survey is to understand the basic situation of older people in terms of diet, behavior and lifestyle, illness condition and so on. In order to effectively track the changes in the ability of ADL, IADL, and COG of the older population, the cohort for the analysis consists of older adults who simultaneously participated in the survey in all four waves of the survey—2008, 2011, 2014, and 2018. After data cleaning, a total of 1930 valid individuals were included in the study sample.

### 2.2. Variable Definitions

#### 2.2.1. Dependent Variables

The health status of the older population, in relation to physical and mental health, is the dependent variable in this study. This paper comprehensively assesses the health status of older adults using the ADL [32], the LADL [33] and COG [34,35]. Specifically, the ability to perform ADL is assessed based on six items: bathing, dressing, indoor transfer, toilet, eating and continence. If the respondents can complete the ADL without assistance, it is coded as 1; otherwise, it is coded as 0, with a score range of 0~6. The IADL evaluation consists of the following eight items: visiting neighbors, shopping, cooking, washing, walking 1 km continuously, lifting 5 kg weights, squatting and standing 3 times continuously, and travelling alone by public transport. If the respondents can complete the IADL by themselves, it is coded as 1; otherwise, it is coded as 0, with a score range of 0~8. The COG of older adults is assessed using the Mini-Mental State Examination (MMSE), which is a comprehensive cognitive assessment tool widely used internationally and with good reliability [36,37]. To be specific, the cognitive ability in this study relates to general ability, reaction ability, attention and calculation ability, memory and language understanding, and self-coordination ability, consisting of 24 items and a total score range of 0 to 30.

In addition, in order to clarify the mechanism of leisure activities for the health status of older adults, this study further defines the ADL, IADL, and COG; scores of ADL/IADL/COG that are less than 6, 8, or 24, respectively, are defined as ADL disorder (yes = 1, no = 0) [3], IADL disorder (yes = 1, no = 0) [3], or cognitive impairment (yes = 1, no = 0) [38], respectively.

#### 2.2.2. Independent Variables

Leisure activity is the independent variable in this research, and the eight assessed items include housework (cooking, taking care of children), outdoor activities (such as tai chi, square dance, etc.), growing flowers or caring for pets, reading books and newspapers, raising poultry or livestock, playing cards or other games (i.e., mahjong), watching TV and listening to radio, and participating in organized social activities. Each item is ranked on a five-level Likert scale, with a score of 1 representing “Almost every day” and 5 representing “no participation.” In this paper, the negative score of the original scale is transformed; thus, the final score of each item is from 1–5. The higher the score, the higher the degree of leisure activities; therefore, the final score range of leisure activities of older adults is 8–40.

#### 2.2.3. Control Variables

Community service refers to the medical treatment, care, and other living services provided by the community for the older population, which includes a total of 8 items. If the service is supported by the community, it will be coded as 1; otherwise, it is coded as 0. Thus, the total score range is 0–8. In addition, gender (male = 1, female = 0), marriage (married = 1, others = 0), region (urban = 1, rural = 0), and health behaviors (smoking = 1, others = 0; alcohol drinking = 1, others = 0) are considered. Details are shown in Table 1.

### 2.3. Methods of Data Analysis

#### 2.3.1. Latent Growth Curve Model

In this paper, MPLUS8.0 is used to construct a latent growth curve model (LGCM) to examine the trajectory of ADL ability, IADL ability, and COG ability of older adults. LGCM is a variant of the structural equation model, which can describe the variation between repeated measurements by assuming latent trajectories. Different from traditional statistical methods, such as analysis of variance with repeated measures, which focus only on group means, LGCM can estimate both group and individual variation during development [39]. LGCM firstly defines two latent variable structures, namely the initial level and the slope. Then, the actual measurements of a variable at different time points are used to estimate the structure of the two latent variables in the model.

#### 2.3.2. Latent Profile Analysis (LPA)

LPA is a development of the traditional factor analysis method, based on the basic assumptions that the probability of various responses of explicit variables can be explained by a few mutually exclusive potential category variables, respectively, and each category has a specific preference for each observed variable; therefore, the heterogeneous group can be further divided into multiple sub-groups. The advantage of this approach is that a large heterogeneous population is classified into multiple categories of small homogeneous groups, making individuals within the same category similar, with large differences between individuals in different categories [40]. This probability-based multivariable classification no longer requires that observed and latent variables are both continuous. In other words, some differences in leisure activity characteristics between individuals are not or are not merely quantitative differences. There may also be a qualitative difference between certain groups. Accordingly, based on the latent classification analysis model, we can deeply explore the classification of leisure activities of the older population.

#### 2.3.3. Panel Binary Regression

In this study, ADL disorder, IADL disorder and COG impairment are dichotomous data, so panel binary regression is used to examine the impact of leisure activities on the health status of older adults. In this study, a different individual $u_{i}$ is often associated with an explanatory variable; thus, the panel binary fixed effect model is used.

## 3. Results

### 3.1. Sample Descriptive Analysis

According to Table 1, the health status of the older population is good overall. The average values of ADL, IADL and COG are 5.97, 7.04, and 26.68, respectively. Correspondingly, the average values of ADL disorder, IADL disorder, and COG impairment are 0.13, 0.48, and 0.20, respectively. The average age of older adults is 75.37 and the proportion of females (50.13%) is slightly higher than males. The household registration is mainly in rural areas (73.38%). The overall education level of the older population is low, with an average education duration of 2.75 years. Furthermore, the proportion of older people who smoke and drink is relatively low (48.03% and 47.85%, respectively). In addition, the majority of older people receive social security, accounting for as much as 81%, and more than half have access to community services.

### 3.2. Trajectories of ADL, IADL, and COG Ability of Older Adults

**Figure 1:**
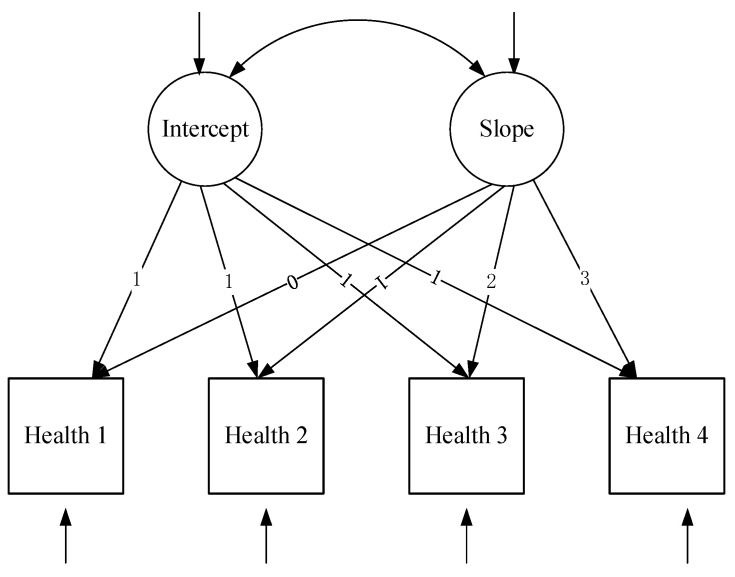
The unconditional linear growth model of the health status of older adults.

**Figure 2:**
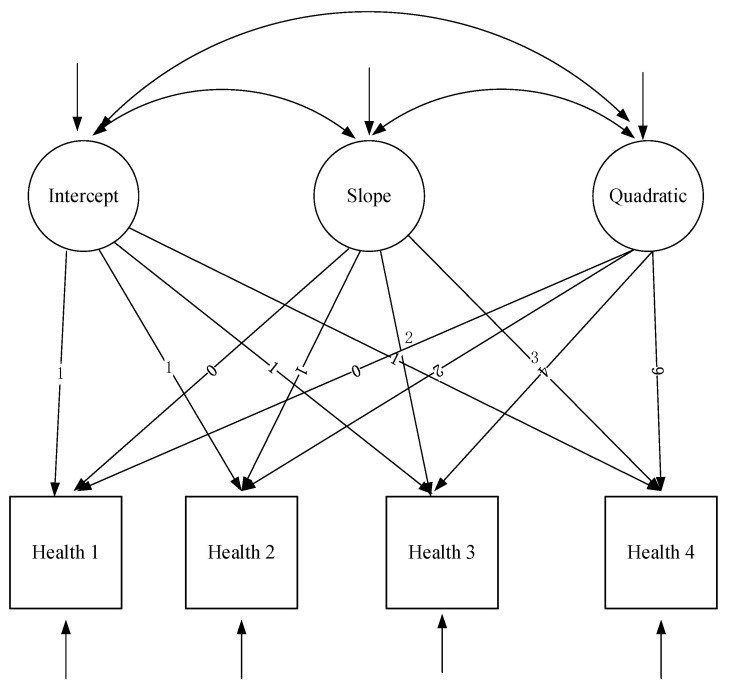
The unconditional nonlinear growth model of health status of older adults.

In order to test the changing trajectory of the health status of older adults, the unconditional linear growth model and nonlinear growth model are constructed, which are shown in Figure 1 and Figure 2, separately. The linear unconditional LGCM only needs to estimate the intercept (α) and slope (*β*). The intercept represents the initial level of the development trend of health status, and all factor loadings are fixed at 1.0. The slope represents the rate of change in the trend of health status, and factor loadings are set to 1.0, 2.0, 3.0, and 4.0, respectively. According to the year of the test, the equation of the first level model is as follows: (1) Health*_ti_* = *α*_0_*_i_* + *β*_1_*_i_*λ*_ti_*+ e*_ti_*. Of these, Health*_ti_* is the health-level score of subject *i* at time *t*; *α*_0_*i* is the intercept of subject *i*, which is the estimated mean of the health status of 2008 in this study; *β*_1_*i* is the slope of subject *i* in this study, which indicates the mean annual change score of health status over the four test periods; *λ_ti_* is the time score, and *e_ti_* is the residual of subject *i* at time *t*. The equations of the second layer are (2) *α*_0_*_i_* = *μ*_00_ + *ξ*_0*i*_; (3) *β*_1_*_i_* = *μ*_10_ + *ξ*_1*i*_. The *μ*_00_ and *μ*_10_ are the mean values of intercept and slope, respectively. The *ξ*_0*i*_ and *ξ*_1*i*_ are residuals of *i* intercept and slope, respectively.

Health*_it_* = *α*_0_*_i_* + *β*_1_*_i_*λ_t_ + e*_it_* (1)

*α*_0_*_i_* = *μ*_00_ + *ξ*_0*i*_ (2)

*β*_1_*_i_* = *μ*_10_ + *ξ*_1*i*_ (3)

The nonlinear unconditional LGCM adds a quadratic term to the linear unconditional growth model; thus, the equation at the first level can be written as follows: (4) Health*_ti_* = *α*_0_*_i_* + *β*_1_*_i_*λ*_ti_* + *β*_2_*_i_*λ*_ti_*^2^ + e*_ti_*. Among them, *β*_1*i*_ is the linear slope and *β*_2*i*_ is the curve slope. The equations of the second layer are as follows: (2) *α*_0_*_i_* = *μ*_00_ + *ξ*_0_; (3) *β*_1*i*_ = *μ*_10_ + *ξ*_1*i*_; (5) *β*_2*i*_ = *μ*_20_ + *ξ*_2*i*_.

Health*_ti_* = *α*_0_*_i_* + *β*_1_*_i_*λ*_ti_* + *β*_2_*_i_*λ*_ti_*^2^ + e*_ti_* (4)

*α*_0_*_i_* = *μ*_00_ + *ξ*_0*i*_ (5)

*β*_1*i*_ = *μ*_10_ + *ξ*_1*i*_ (6)

*β*_2*i*_ = *μ*_20_ + *ξ*_2*i*_ (7)

Similarly, the method of LGCM is applied to leisure activities. Accordingly, the unconditional linear and nonlinear LA models are regarded as model 1 and model 2, separately. Furthermore, the unconditional linear and nonlinear ADL models are regarded as model 3 and model 4, separately. Similarly, the unconditional linear and nonlinear IADL models are regarded as model 5 and model 6 and the unconditional linear and nonlinear COG models are regarded as model 7 and model 8, respectively.

From Table 2, it is indicated that the fitting effect of the nonlinear model is better than that of the linear model in LA, ADL, IADL, and COG, and there are significant differences among the models. To be specific, for model 1, Chi-square = 557, *df* = 5, CFI = 0.563, RMSEA = 0.239, and SRMR = 0.104 for the linear model of leisure activity. For model 2, Chi-square = 36.7, *df* = 1, CFI = 0.972, RMSEA = 0.136, and SRMR =0.028 for the nonlinear model of leisure activity. In addition, the fitting effects of the two models were compared, and following result was obtained: $△\chi^{2}$/△df = 520.3 (4), *p* < 0.05, indicating that the fitting effect of the leisure activity of the nonlinear model (model 2) is significantly better than that of the linear model (model 1). Similarly, for model 3, Chi-square= 115, *df* = 5, CFI = 0.624, RMSEA = 0.107, and SRMR = 0.057 for the linear model of ADL. For model 4, Chi-square = 53.6, *df* = 1, CFI = 0.821, RMSEA = 0.165, and SRMR = 0.039 for the nonlinear model of ADL. In addition, the fitting effects of the two models are compared and the obtained result was as follows: $△\chi^{2}$/△df = 61.4 (4), *p* < 0.05, indicating that the fitting effect of the ADL nonlinear model (model 4) is significantly better than that of the linear model (model 3). Similarly, for model 5, Chi-square = 225, *df* = 5, CFI = 0.888, RMSEA = 0.151, and SRMR = 0.061 for the linear model of IADL. For model 6, Chi-square = 19.9, *df* = 1, CFI = 0.99, RMSEA = 0.099, and SRMR = 0.018 for the nonlinear model of IADL. In addition, the fitting effects of the two models are compared and the obtained result was as follows: $△\chi^{2}$/△df = 205.1 (4), *p* < 0.05, which suggests that the fitting effect of the IADL nonlinear model (model 6) is superior to the linear model (model 5). Likewise, for model 7, Chi-square = 233, *df* = 5, CFI = 0.824, RMSEA = 0.154, SRMR = 0.095 for the linear model of COG. For model 8, Chi-square = 19.1, *df* = 1, CFI = 0.986, RMSEA = 0.097, SRMR = 0.019 for the nonlinear model of COG. Additionally, the fitting effects of the two models are compared, and the following result was obtained: $△\chi^{2}$/△df = 213.9 (4), *p* < 0.05, which suggests that the fitting effect of the COG nonlinear model (model 8) is significantly better than that of the linear model (model 7). Consequently, from 2008 to 2018, the leisure activity and the abilities of ADL, IADL, and COG of the older population show a significant trend of nonlinear changes.

**Figure 3:**
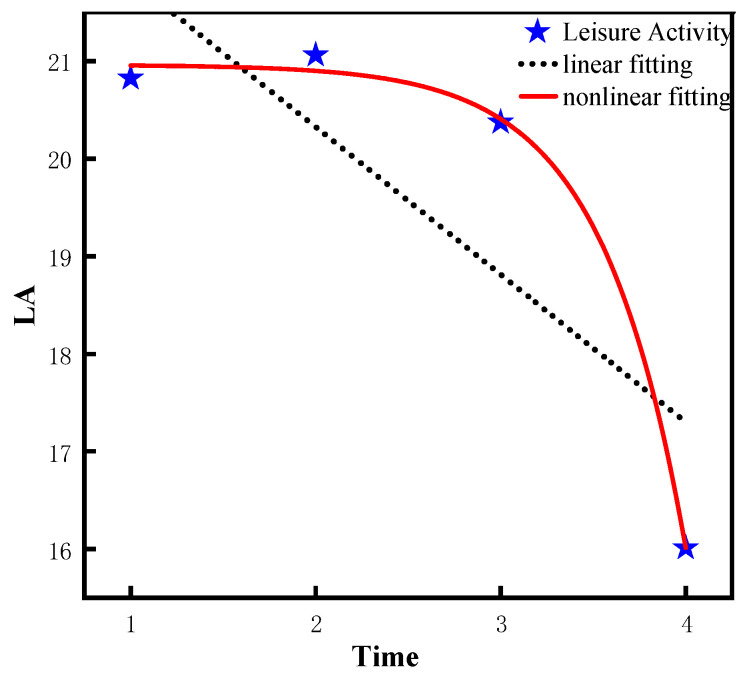
Trajectory of leisure activity of older adults.

**Figure 4:**
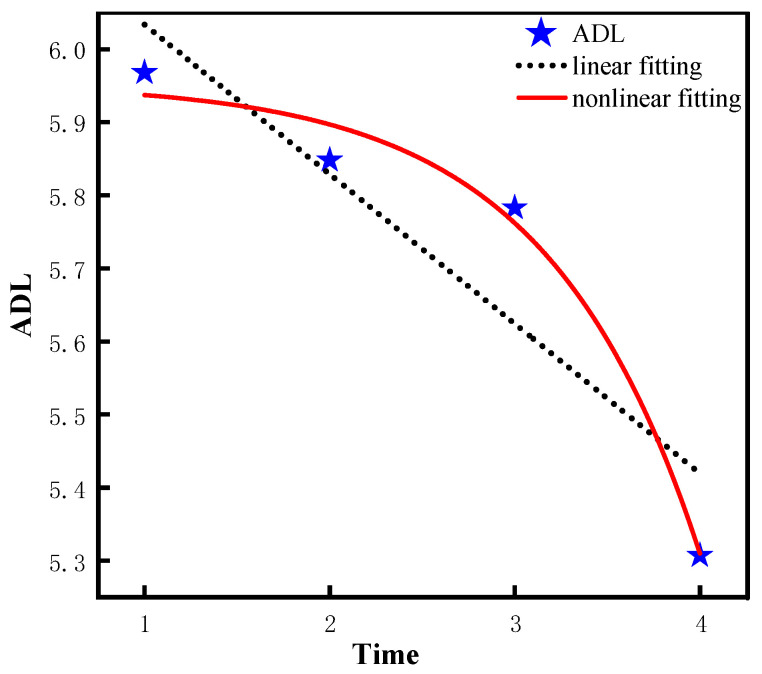
Trajectory of ADL ability of older adults.

**Figure 5:**
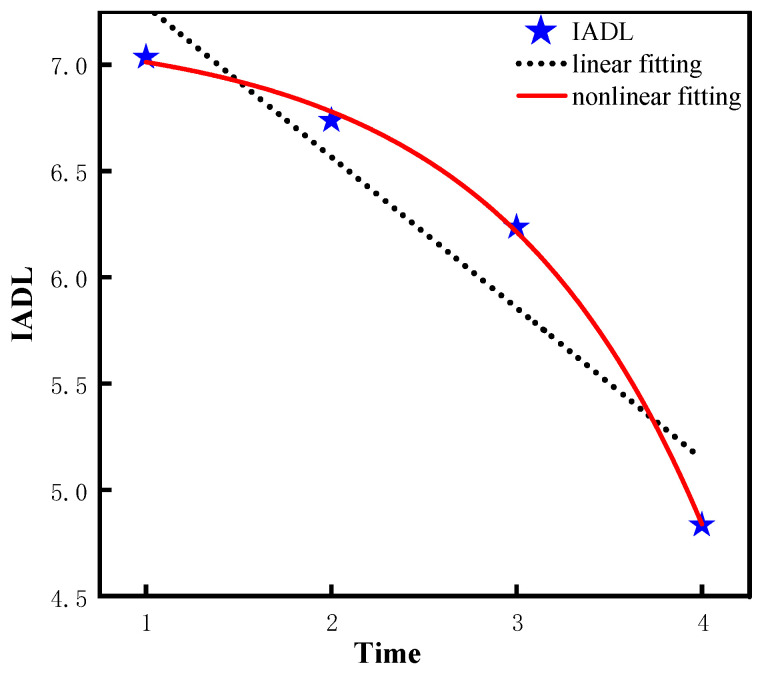
Trajectory of IADL ability of older adults.

**Figure 6:**
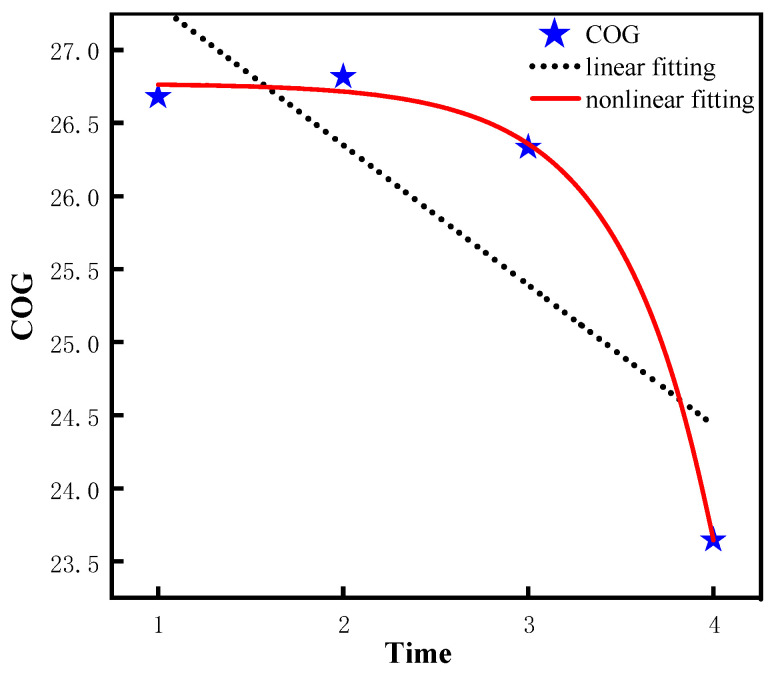
Trajectory of COG ability of older adults.

According to Table 3, the quadratic slope of the leisure activity nonlinear model (model 2) is −1.16 (*p* < 0.001), indicating the leisure activity decreases significantly from 2008 to 2018. Similarly, the quadratic slope of the ADL nonlinear model (model 4) is −0.06 (*p* < 0.001, indicating the ADL ability decreases significantly from 2008 to 2018. In addition, the variance of intercept = 0.10 is significantly greater than 0 (*p* < 0.001, which suggests that there are significant differences in the initial ADL ability among different individuals. Similarly, the quadratic slope of the IADL nonlinear model (model 6) is−0.26 (*p* < 0.001), which suggests the IADL ability decreases significantly from 2008 to 2018. In addition, the intercept variance =1.88 is significantly greater than 0 (*p* < 0.001), and the variance of the quadratic slope =0.14 is significantly greater than 0 (*p* < 0.001), which indicates that the initial ability and the rate of change of IADL are significantly different among different older adults. Similarly, the quadratic slope of COG nonlinear model (model 8) is −0.68 (*p* < 0.001), which suggests that the COG ability decreases significantly from 2008 to 2018. In addition, the intercept variation =9.60 is significantly greater than 0 (*p* < 0.001), and the quadratic slope variation = 0.62 is significantly greater than 0 (*p* < 0.05), suggesting that there are significant differences in the initial ability and the rate of change of COG among different individuals. Lastly, the difference of the fitting effect between the linear model and non-linear model is visualized by using the graphic method in this study, as shown in Figure 3, Figure 4, Figure 5 and Figure 6.

### 3.3. Analysis of the Effect of Leisure Activities on the Health Status of the Older Population

According to the above analysis, the ADL, IADL, and COG of the older population all show a declining trend over time. Considering this, it is essential to determine how best to prevent these declines. Therefore, the panel data from 2008 to 2018 are used to analyze the impact of leisure activities on ADL disorder, IADL disorder, and COG impairment. Because the dependent variables of this study are all dichotomous variables, the panel binary regression model (xtlogit) is used in this paper. Moreover, due to the missing variables that do not change over time, the fixed-effects model (FE) is considered in this study. In addition, the method of Jackknife is used to reduce the standard error of random error. Details are shown in Table 4.

According to Table 4, leisure activities have a significant inhibitory effect on ADL disorder, IADL disorder and cognitive impairment. Specifically, a unit increase in leisure activity will result in a ($1-e^{-0.08}$) * 100 = 7.69% reduction in the probability of ADL disorder, with 95% confidence interval [0.9015, 0.9475]; a ($1-e^{-0.08}$) * 100 = 7.69% reduction in the probability of IADL impairment, with 95% confidence interval [0.9062, 0.9360]; and a ($1-e^{-0.11}$) * 100 = 10.42% reduction probability of cognitive impairment, with 95% confidence interval [0.8793, 0.9125]. In addition, age has a significant positive effect on ADL disorder, IADL disorder, and COG impairment. To be specific, as age increases, so does the probability of the older population suffering from ADL disorder, IADL disorder, and COG impairment. Furthermore, community service can significantly reduce the probability of COG impairment in the older population.

### 3.4. Latent Classification Analysis of Leisure Activities

In this study, LPA is used to classify the older population according to their choice of leisure activities. To be specific, it is considered that a posteriori probability value of greater than 0.8 is a good classification scheme [41]. In addition, the greater the entropy, the better the classification effect, with a general entropy of <0.6 indicating <80% of cases being correctly classified and an entropy of >0.8 indicating that >90% of cases are correctly classified [42]. Finally, the indicators of AIC/BIC/aBIC are essential to determine the appropriate classification. Specifically, the smaller the value, the better. In order to determine the best classification effect, the older population are classified into 1, 2, 3, and 4 categories separately, and the fitted information indexes of different classification types are compared, as shown in Table 5.

**Figure 7:**
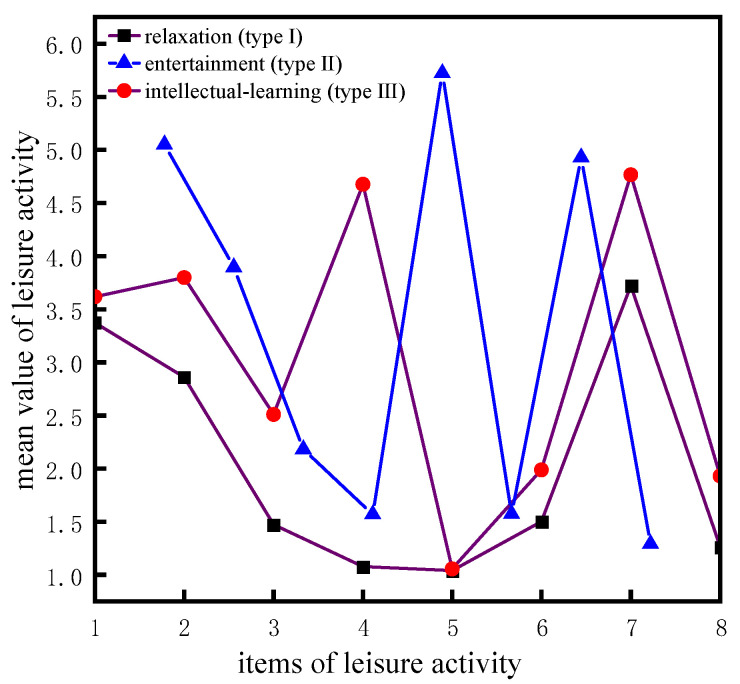
A latent profile analysis of leisure activities.

According to Table 5, it is suitable to classify older adults into two or three categories. In addition, combined with the values of AIC, BIC, and ABIC, it is more appropriate to classify older adults into three categories, each accounting for 56.684%, 30.363%, and 12.953% of the population, respectively. Moreover, the posterior probability value of each category is 0.995, 0.994, and 0.988, which are all greater than 0.8, which indicates that 99.5% of the individuals belonging to type I are actually classified as type I. Similarly, 99.4% and 98.8% of the individuals belonging to type II and III are actually classified as type II and III. The results of classification are shown in Figure 7.

It can be indicated that type I older adults spend most of their time watching tv or listening to the radio and doing housework (item 7 and item 1). For type II older people, outdoor activity, keeping pets, and playing cards or mahjong are the main activities in their free time (item 2, item 5, and item 6). For type III older adults, they prefer to read books and watch tv or listen to the radio when they have free time (item 4 and item 7). Accordingly, we name type I, type II, and type III as relaxation, entertainment, and intellectual-learning, respectively.

### 3.5. Effect of Latent Classifications of Leisure Activity on the Health Status of Older Adults

According to the analysis of LPA of leisure activities, the older population can be classified into relaxation (type I), entertainment (type II), and intellectual-learning (type III). Based on this classification, a one-way ANOVA is used to test whether the health status of the older population with different types of leisure activities significantly different. The results are shown in Table 6.

From Table 6, it can be seen that there is a significant difference in the rate of ADL disorder among the older population with different types of leisure activities from 2008 to 2018. Specifically, from 2008 to 2018, the rate of ADL disorder is significantly lower in older adults who prefer entertainment (type II) over relaxation (type I). For 2018, the prevalence of ADL disorder was found to be significantly lower in the older adults with intellectual-learning (type III) than the older population with relaxation (type I).

Similarly, from 2008 to 2018, there are significant differences in the rate of IADL disorder among the older population with different types of leisure activities. To be specific, from 2008 to 2018, the disorder rate of the IADL in older adults with entertainment (type II) is significantly lower than for the older population with relaxation (type I), and the IADL disorder rate of the older population with intellectual-learning (type III) is significantly lower than the older population with relaxation (type I).

In addition, from 2008 to 2018, it is indicated that there are significant differences in cognitive impairment rates among older people with different types of leisure activities. Specifically, from 2008 to 2018, the cognitive impairment rate of older adults who engaged in recreational activities (type II) was found to be significantly lower than for the older population with relaxation activities (type I), and the cognitive impairment rate of older people who engage in intellectual-learning activities (type III) is significantly lower than older people with relaxation (type I). Moreover, except for 2014, the cognitive impairment rate of the older population with intellectual-learning (type III) was found to be significantly lower than older adults with entertainment (type II).

### 3.6. Moderating Effect of Age on Leisure Activities on ADL Disorder, IADL Disorder, and COG Impairment

In order to explore whether the relationship between leisure activities and health level is affected by age, according to the standard of the WHO, older people aged 60–74 are classified as the low-aged group, those aged 75–89 are classified as a middle-aged group, and those who are 90 years older are classified as the high-aged group [43]. In view of the above, a group regression is performed (results are shown in Table 7) and a test for difference in the regression coefficients for the different groups is performed, using bootstrap. Due to space limitations, this study only reports the grouping regression coefficient of leisure activities on health status of older adults, the details of which are shown in Table 8.

According to Table 7, leisure activities have no significant inhibitory effect on ADL disorder in the low-age group (β = −0.04), but do impart a significant inhibitory effect on ADL disorder in the middle-age group (β = −0.06 **) and high-aged group (β = −0.18 ***). To test whether the effects of leisure activities on ADL disorder are heterogeneous across age groups, a Fischer’s Permutation test is further conducted on the grouping regression coefficients, which is shown in Table 8. Consequently, the effect of leisure activities on ADL disorder is significantly different between the young-aged and the middle-aged older adults (*p* < 0.05); specifically, engaging in leisure activities has a better effect on the middle-aged group. Furthermore, there is no significant difference in the effect of leisure activities on ADL disorder between the middle-aged and the high-aged groups (*p* > 0.05), but there is a significant difference in the effect of leisure activities between the low-aged and the high-aged groups (*p* < 0.001); specifically, engaging in leisure activities has a better effect on the high-aged group.

Similarly, leisure activities have a significant inhibitory effect on IADL disorder for the low-aged group (β = −0.01 *), middle-aged group (β = −0.08 **), and high-aged groups (β = −0.20 ***). Moreover, from Table 8, the effect of leisure activities on IADL disorder can be seen to be significantly different between the low-aged and the middle-aged group (*p* < 0.05), specifically indicating that the inhibition effect of leisure activities on IADL disorder is more obvious in the middle-aged people. In addition, the effect of leisure activities on ADL disorder is not significantly different between the middle-aged and the high-aged group (*p* > 0.05), but there is a significant difference between the low-aged and the high-aged group (*p* < 0.001), specifically, showing a greater effect in the high-aged group.

Finally, leisure activities were found to have a significant inhibitory effect on COG impairment in the low-aged (β = −0.10 *), middle-aged (β = −0.11 **), and high-aged group (β = −0.14 ***). As can be seen from Table 8, the effect of leisure activities on COG impairment is significantly different between the low-aged and the middle-aged group (*p* < 0.05), and a greater effect on middle-aged older people is noticeable. Additionally, there is no significant difference between the middle-aged and the high-aged group in the effect of leisure activities on COG impairment (*p* > 0.05), while there is a significant difference between the low-aged and the high-aged group (*p* < 0.05), indicating a greater effect on the high-aged group.

### 3.7. Robustness Test

In order to verify the validity of the conclusion that leisure activities affect the health status of the older population, this paper used the level of ADL ability, the level of IADL ability and the level of COG ability to replace the ADL disorder, IADL disorder, and COG impairment, respectively. Because the health status of older people (ADL level, IADL level and COG level) is provided as continuous data, panel regression for robustness is used. It can be drawn from Table 9 that the higher the level of leisure activity, the higher the level of ADL (β = 0.03 ***), IADL (β = 0.13 ***) and COG (β = 0.22 ***), which suggests that leisure activities can effectively improve the health level of the older population. In other words, leisure activities can effectively decrease the probability of ADL disorder, IADL disorder, and COG impairment in older adults, which means the conclusion of this study is convincing.

## 4. Discussion

In this section, we mainly study the changing trajectory of LA, ADL, IADL, and COG ability of older adults. In addition, the effects of leisure activities on the health status of older adults are discussed. Furthermore, the effect of the latent profile analysis of leisure activities on the health status of older adults is included. Finally, we discuss the moderating effect of age on the relationship between leisure activities and health status.

### 4.1. Changing Trajectory of LA, ADL, IADL, and COG Ability of Older Adults

According to the results of the LGCM, the leisure activities and the abilities of ADL, IADL, and COG of the older population in the CLHLS show a significant nonlinear decline trend overtime, which is in line with existing research findings on cognitive ability [44,45]. For leisure activities, the result was consistent with the previous study [46]. With the increasing age of older adults, they gradually suffer from arthritis and other diseases, which severely limits their physical and recreational activities [47]. In relation to health status, from the biological point of view, with increasing age, muscles gradually lose elasticity and presents symptoms such as muscle weakness and muscle atrophy, which affects the health standard of the older population [48]. In addition, older people are susceptible to a variety of chronic diseases, such as hypertension, diabetes, cerebrovascular disease, and diabetes, which will affect the health status of older adults and thus leads to the decline of ADL ability and IADL ability in the older population. Moreover, research supports that the relationship between physical health and cognitive ability is bidirectional [49,50], which means that when the physical health level of the older population tends to decline over time, so does their cognitive ability. In other words, the deterioration of the physical health level of older adults will also lead to their cognitive decline. In addition, according to the life cycle theory, the decline in physical function accelerates over time as older people reach the end of their life cycle [51]. Furthermore, the cognition ability of older adults declined at an accelerated rate later in the life cycle [52].

### 4.2. Effects of Leisure Activities on ADL and IADL Disorder, and COG Impairment

According to Table 4, leisure activities can significantly decrease the rate of ADL disorder, IADL disorder, and cognitive impairment in the older population from 2008 to 2018. From a biological point of view, leisure activities, especially outdoor activities, can activate endocrine, immune, and central nervous system mechanisms that affect the metabolic system of the heart and affect physical performance, which triggers a multi-system biological response that affects the health level of the older population, resulting in an increase in ADL and IADL abilities [53]. As we know, the process of leisure activities often involves physical exercise [54,55]; therefore, leisure activities can promote ADL ability and IADL ability, thus reducing the probability of suffering from ADL inability and IADL inability among older adults. Furthermore, entertainment leisure activities, such as square dance, planting flowers, and caring for pets, may also involve some physical exercise, thus reducing the probability of ADL inability and IADL disorder. In addition, participating in leisure activities often motivates individuals to engage in healthy behaviors and/or to reduce unhealthy behaviors, thereby ensuring an improvement in individuals’ level of health, and improving the ADL and IADL abilities of older persons [53]. In addition, leisure activities can lead to changes in behavioral mechanisms, such as those related to the development of health habits, which in turn influence behavioral decision-making, behavioral drivers, and behavioral development. These factors ultimately promote healthy behaviors, which affects health, as well as ADL ability and IADL in older adults [53].

Additionally, leisure activities in this study are shown to have a significant promoting effect on cognitive ability, which is consistent with the findings of previous studies [56,57]. According to the theory of cognitive reservation, intellectual-learning leisure activities such as reading and listening to radio can stimulate older people to keep learning [58,59], thus effectively preventing cognitive decline. Moreover, relaxation leisure activities stimulate the brain to promote cognitive improvement in older adults by improving overall health [60]. Additionally, some entertainment leisure activities, such as playing mahjong, chess, and card activities, require individuals to engage the part of the brain that helps to maintain an active state, which is beneficial to the cognitive ability of older adults. Finally, leisure activities can effectively improve cognition by promoting the physical health [30,60] and the subjective well-being of older people [8].

### 4.3. Effect of Latent Profile Analysis of Leisure Activities on the Health Status of Older Adults

According to the LPA of leisure activities, the older population are classified into the following three categories: relaxation (Type I), entertainment (Type II), and intellectual-learning (Type III). For the ADL ability and IADL abilities, from 2008 to 2018, the rate of ADL disorder and IADL disorder in older adults of Type II is significantly lower than counterparts of Type I. One possible explanation is that entertainment leisure activities, such as outdoor activities, usually involve physical exercise, which is indicated to improve the health status of older adults [61,62], e.g., resistance training [63]. In contrast, relaxation leisure activities, such as watching TV or listening to radio, usually do not require older adults to engage in physical movement. Accordingly, compared with older people of Type I, the older population of Type II have a better health status and thus a lower probability of ADL and IADL disorder.

For cognitive ability, from 2008 to 2018, the cognitive impairment rate of the older population of Type II and Type III is significantly lower than the counterpart of Type I. In addition, in 2008, 2011, and 2018, the cognitive impairment rate of the older adults of Type III is significantly lower than older adults of Type II. Intellectual-learning leisure activities often require individuals to mobilize the brain’s neural mechanisms to stimulate the brain in a state of continuous operation, which is in line with the theory of “use it or lose it” [64,65]. For instance, it is indicated that cognitive training intervention can maintain or improve cognition for older adults [66,67]. Therefore, the effect of intellectual leisure activities on cognitive ability is the most significant.

### 4.4. Moderating Effect of Age on the Relationship between Leisure Activities and Health Status

From Table 7, it can be considered that leisure activities have no significant effect on the probability of ADL disorder in the low-aged group, but have a significant inhibitory effect on the middle-aged group and the high-aged group. In addition, leisure activities significantly alleviated the probability of IADL disorder and cognitive impairment in the low-aged, middle-aged, and high-aged group. To compare the difference in the effect of leisure activities on health status among different age groups, the difference of the coefficient of grouping regression was tested and the results show that the inhibitory effects of leisure activities on ADL disorder, IADL disorder, and cognitive impairment in the low-aged group is significantly lower than those in the middle-aged and high-aged group. One alternative explanation is that low-aged older people tend to be healthier, including in terms of ADL, IADL, and COG levels, than middle-aged and high-aged older people, according to the life-cycle theory [68]. Accordingly, the effect of leisure activities on ADL disorder, IADL disorder, and cognitive impairment is not significant in low-aged older people. For middle-aged older people and high-aged older people, due to physical function degradation, their ADL ability, IADL ability, and cognitive ability are also relatively low [69] and declines rapidly over time. Thus, due to the stimulation of leisure activities, their ADL ability, IADL ability and cognitive ability will be obviously improved. As a result, the inhibitory effect of leisure activities on ADL disorder, IADL disorder and cognitive impairment in the low-aged group is significantly lower than in the middle-age group and high-aged group.

## 5. Contributions and Limitations

Firstly, this study uses the LGCM to study the trajectory of ADL ability and IADL ability in older adults. Secondly, this study analyzes the effects of leisure activities on ADL disorder and IADL disorder based on the four survey waves (2008, 2011, 2014, 2018) of the CLHLS. Thirdly, previously, the classification of leisure activities was based on subjective cognition and lacked scientific rigor, while LPA was used to classify the older population according to their choice of leisure activities in this study. Specifically, the older population in the survey are scientifically classified into three categories of leisure activities, namely the type of relaxation, entertainment, and intellectual-learning, respectively. These are the innovations and contributions of this study.

However, the study is subject to some limitations. The latest data in the public database used in this study were from 2018, and the data are lagging, which is a limitation of this study. In addition, this study only considers the impact of the number of leisure activities on the health status of the older population. To better understand the relationship between leisure activities and the health status of older adults, the frequency and duration of leisure activities should be taken into account; these factors were not accounted for in this study.

## 6. Implications

From the above analysis, older adults should be encouraged to take part in leisure activities, especially middle-aged and high-aged older adults. In addition, for the types of leisure activities, older adults should balance all types of leisure activities, e.g., relaxation, entertainment, and intellectual-learning. Finally, intellectual-learning leisure activities should be more frequently practiced, as they are more beneficial to the improvement of health status of older adults.

## 7. Conclusions

From the above analysis, the following conclusions are derived:

① From 2008–2018, the leisure activities, ADL ability, IADL ability, and cognitive ability of the older population all show a significant nonlinear decline trend over time.
② Overall, leisure activities have a significant positive impact on the health level of the older population. Specifically, leisure activities are suggested to decrease the probability of ADL disorder, IADL disorder, and the cognitive impairment of older adults.
③ The older population are classified according to engagement in the following three types of activity—relaxation, entertainment, and intellectual-learning—according to the latent profile analysis (LPA) conducted, and there are significant differences in the rates of ADL disorder, IADL disorder, and cognitive impairment among different types of older adults. In general, the rates of ADL disorder, IADL disorder, and cognitive impairment in older people with intellectual-learning leisure activities (Type III) are successively lower than in older people with entertainment (Type II) and relaxation leisure activities (Type I).
④ The effects of leisure activities on the rate of ADL disorder, IADL disorder and cognitive impairment are significantly different in different age groups. Specifically, the inhibitory effects of leisure activities on ADL disorder, IADL disorder and cognitive impairment are greater in the middle-aged and high-aged older groups; however, there is no significant difference between the middle-aged and the high-aged groups.

## Figures and Tables

**Table 1:** Descriptive statistical analysis of samples in 2008.

Variables	Definition	Mean (SD)
ADL	The total score of ADL (0~6)	5.97 (0.29)
IADL	The total score of IADL (0~8)	7.04 (1.82)
COG	The total score of cognitive ability (0~30)	26.68 (−4.53)
ADL disorder	The total score of ADL < 6 = 1; other = 0	0.13 (−0.33)
IADL disorder	The total score of IADL < 8 = 1; other = 0	0.48 (−50)
COG impairment	The total score of COG < 24 = 1; other = 0	0.20 (−40)
Age	The true age of older adults in 2008 (61~108)	75.37 (−28)
Gender	male = 1; female = 0	0.46 (0.50)
Region	urban = 1; rural = 0	0.08 (0.27)
Living arrangement	living with family = 1; living alone = 2; living in an institution = 3	1.18 (0.40)
Education	The years of schooling (0~20)	2.75 (3.61)
Marriage	married = 1; others = 0	0.60 (0.49)
Smoking	smoked = 1; others = 0	0.36 (0.48)
Alcohol drinking	drunk = 1; others = 0	0.35 (0.48)
Income	Ln(salary)	9.08 (1.29)
Social security	yes = 1; no = 0	0.81 (0.39)
Community service	The total score of community service (0~8)	0.55 (1.28)

SD denotes standard deviation.

**Table 2:** Model fitting effect.

Variables	Models	*χ^2^*(*df*)	*p*	CFI	RMSEA	SRMR	Model Comparison	*p*
LA	Linear model (1)	557 (5)	<0.001	0.563	0.239	0.104	$△\chi^{2}$/△df = 520.3 (4)	<0.005
	Nonlinear model (2)	36.7 (1)	<0.001	0.972	0.136	0.028		
ADL	Linear model (3)	115 (5)	<0.001	0.624	0.107	0.057	$△\chi^{2}$/△df = 61.4 (4)	<0.005
	Nonlinear model (4)	53.6 (1)	<0.001	0.821	0.165	0.039		
IADL	Linear model (5)	225 (5)	<0.001	0.888	0.151	0.061	$△\chi^{2}$/△df = 205.1 (4)	<0.005
	Nonlinear model (6)	19.9 (1)	<0.001	0.99	0.099	0.018		
COG	Linear model (7)	233 (5)	<0.001	0.824	0.154	0.095	$△\chi^{2}$/△df = 213.9 (4)	<0.005
	Nonlinear model (8)	19.1 (1)	<0.001	0.986	0.097	0.019		

**Table 3:** Model parameter values.

Variables	Means of Growth Factors			Variance of Growth Factors		
	Intercept	Slope	Quadratic	Intercept	Slope	Quadratic
LA	21.90 ***	−1.61 ***		8.27 ***	0.22	
	20.66 ***	1.98 ***	−1.16 ***	4.61	0.78	0.20
ADL	5.97 ***	−0.13 ***		0.04 ***	0.05 ***	
	5.97 ***	−0.01	−0.06 ***	0.10 ***	0.13 **	0.01
IADL	7.19 ***	−0.62 ***		1.52 ***	0.24 ***	
	7.01 ***	0.08	−0.26 ***	1.88 ***	1.13 **	0.14 ***
COG	27.20 ***	−0.69 ***		3.96 ***	0.96 ***	
	26.61 ***	1.12 ***	−0.68 ***	9.60 ***	7.29 **	0.62 *

* *p* < 0.05, ** *p* < 0.01, *** *p* < 0.001.

**Table 4:** The effect of leisure activities on ADL disorder, IADL disorder, and cognitive impairment.

Variables	ADL Disorder			IADL Disorder			COG Impairment		
	B	OR	95% CI	B	OR	95% CI	B	OR	95% CI
Leisure activities	−0.08 ***	0.92 ***	[0.9015, 0.9475]	−0.08 ***	0.92 ***	[0.9062, 0.9360]	−0.11 ***	0.90 ***	[0.8793, 0.9125]
	(0.01)	(0.01)		(0.01)	(0.01)		(0.01)	(0.01)	
Community service	−0.02	0.98	[0.9210, 1.0439]	0.00	1.00	[0.9574, 1.0462]	−0.07 *	0.93 *	[0.8827, 0.9843]
	(0.03)	(0.03)		(0.02)	(0.02)		(0.03)	(0.03)	
Age	0.24 ***	1.27 ***	[1.2332, 1.3176]	0.20 ***	1.22 ***	[1.1880, 1.2457]	0.08 ***	1.09 ***	[1.0611, 1.1139]
	(0.02)	(0.02)		(0.01)	(0.02)		(0.01)	(0.01)	
Region	−0.21	0.81	[0.4606, 1.4219]	−0.25	0.78	[0.5113, 1.1959]	−0.13	0.88	[0.5802, 1.3420]
	(0.29)	(0.23)		(0.22)	(0.17)		(0.21)	(0.19)	
Living arrangement	−0.34 *	0.71 *	[0.5367, 0.9430]	−0.24 *	0.78 *	[0.6171, 0.9939]	−0.03	0.97	[0.7651, 1.2393]
	(0.14)	(0.10)		(0.12)	(0.10)		(0.12)	(0.12)	
Years of education	0.04	1.04	[0.9841, 1.1089]	−0.02	0.98	[0.9441, 1.0267]	−0.03	0.97	[0.9157, 1.0288]
	(0.03)	(0.03)		(0.02)	(0.02)		(0.03)	(0.03)	
Marriage	−0.06	0.95	[0.6158, 1.4530]	−0.38 *	0.68 *	[0.5025, 0.9287]	−0.18	0.84	[0.6005, 1.1622]
	(0.22)	(0.21)		(0.16)	(0.12)		(0.17)	(0.14)	
Smoking	0.02	1.02	[0.6683, 1.5608]	0.09	1.10	[0.8258, 1.4595]	0.03	1.03	[0.7166, 1.4892]
	(0.22)	(0.22)		(0.15)	(0.16)		(0.19)	(0.19)	
Alcohol drinking	0.13	1.14	[0.7836, 1.6497]	−0.08	0.92	[0.7194, 1.1789]	−0.02	0.98	[0.7326, 1.3182]
	(0.19)	(0.22)		(0.13)	(0.12)		(0.15)	(0.15)	
Ln(income)	−0.04	0.96	[0.9013, 1.0290]	−0.04	0.96	[0.9194, 1.0064]	0.02	1.02	[0.9709, 1.0696]
	(0.03)	(0.03)		(0.02)	(0.02)		(0.03)	(0.03)	
Social security	0.02	1.02	[0.7155, 1.4552]	0.16	1.17	[0.9190, 1.4999]	−0.08	0.92	[0.6959, 1.2197]
	(0.18)	(0.18)		(0.13)	(0.15)		(0.14)	(0.13)	

* *p* < 0.05, *** *p* < 0.001. Standard errors are in parentheses.

**Table 5:** Summary of fitting information of latent category analysis.

Model	k	AIC	BIC	aBIC	Entropy	LMR	BLRT	Class Probabilities
1 C	16	223,666.097	223,777.323	223,726.478	——	——	——	1
2 C	25	205,586.142	205,759.931	205,680.487	0.991	0.0000	0.0000	0.69262/0.30738
3 C	34	200,001.442	200,237.795	200,129.750	0.987	0.0000	0.0000	0.56684/0.30363/0.12953
4 C	43	200,464.376	200,763.293	200,626.648	0.984	0.9997	1.0000	0.54352/0.27241/0.10544/0.07863

**Table 6:** Difference analysis of the impact of latent classification of leisure activities on the health status of older adults.

Types of Health	Year	Type I (Relaxation)	Type II (Entertainment)	Type III (Intellectual-Learning)	*p*	*p (1 vs. 2)*	*p (2 vs. 3)*	*p (1 vs. 3)*
ADL disorder	2008	0.03	0.00	0.02	0.0008	<0.001	0.455	0.752
	2011	0.12	0.07	0.09	0.0031	0.002	0.984	0.436
	2014	0.15	0.10	0.10	0.0016	0.004	1.000	0.056
	2018	0.31	0.14	0.12	<0.001	<0.001	1.000	<0.001
IADL disorder	2008	0.42	0.24	0.18	<0.001	<0.001	0.284	<0.001
	2011	0.52	0.32	0.25	<0.001	<0.001	0.111	<0.001
	2014	0.60	0.38	0.37	<0.001	<0.001	1.000	<0.001
	2018	0.75	0.51	0.51	<0.001	<0.001	1.000	<0.001
COG impairment	2008	0.22	0.11	0.03	<0.001	<0.001	0.014	<0.001
	2011	0.19	0.13	0.03	<0.001	0.002	0.000	<0.001
	2014	0.23	0.11	0.06	<0.001	<0.001	0.252	<0.001
	2018	0.39	0.18	0.08	<0.001	<0.001	0.044	<0.001

**Table 7:** The effect of leisure activities on the health of older adults in different age groups.

Variables	Low-Aged (60~74)	Middle-Aged (75~89)	High-Aged (≥90)
ADL disorder	0.04	−0.06 **	−0.18 ***
IADL disorder	−0.01 *	−0.08 ***	−0.20 ***
COG impairment	−0.10 *	−0.11 ***	−0.14 ***

* *p* < 0.05, ** *p* < 0.01, *** *p* < 0.001.

**Table 8:** Analysis of the difference of regression coefficient by group.

Variables	*p* (Low-Aged vs. Middle Aged)	*p* (Middle-Aged vs. High Aged)	*p* (Low-Aged vs. High-Aged)
ADL disorder	0.030	0.360	<0.001
IADL disorder	0.010	0.120	<0.001
COG impairment	0.040	0.390	0.020

**Table 9:** Robustness test.

	(1)	(2)	(3)
Variables	ADL ability	IADL ability	COG ability
Leisure activities	0.03 ***	0.13 ***	0.22 ***
	(0.003)	(0.006)	(0.014)

*** *p* < 0.001.

## Data Availability

Data derived from public domain resources.
